# Factors Affecting SARS-CoV-2 IgG Production after Vaccination and/or Disease: A Large-Scale Seroprevalence Study

**DOI:** 10.3390/vaccines11101615

**Published:** 2023-10-19

**Authors:** Tanja Karl, Anja Schuster, Lea Maria Stangassinger, Tanja Stiboller, Janne Cadamuro, Gertie Janneke Oostingh

**Affiliations:** 1 Department of Health Sciences, Biomedical Sciences, Salzburg University of Applied Sciences, 5412 Puch/Salzburg, Austria; anja.schuster@fh-salzburg.ac.at (A.S.); lea.stangassinger@fh-salzburg.ac.at (L.M.S.); tanjastibo2906@gmail.com (T.S.); geja.oostingh@fh-salzburg.ac.at (G.J.O.); 2 Research Program of Medical Sciences, Paracelsus Medical University, 5020 Salzburg, Austria; 3 Department of Laboratory Medicine, Paracelsus Medical University, 5020 Salzburg, Austria; j.cadamuro@salk.at

**Keywords:** COVID-19, SARS-CoV-2-specific antibodies, influencing factors, seroprevalence study

## Abstract

This study aimed at identifying factors influencing SARS-CoV-2-specific IgG antibody levels after vaccination and/or infection. Between January 2022 and March 2023, 2000 adults (≥18 years, Salzburg, Austria) participated in this population-based seroprevalence study by providing 3 mL of blood to detect SARS-CoV-2-specific IgG antibodies using an anti-SARS-CoV-2 IgG quantitative assay and by completing a self-designed questionnaire including anthropometric factors, vaccination information, and medical history. For 77 of the participants, a time-course study up to 24 weeks post vaccination or quarantine end was performed. Convalescent-only subjects had the lowest median antibody titer (65.6 BAU/mL) compared to vaccinated and hybrid immunized subjects (*p*-value < 0.0001) The type of vaccine as well as vaccine combinations significantly influenced the levels of SARS-CoV-2 spike-protein-specific IgG, ranging from a median antibody level of 770.5 BAU/mL in subjects who were vaccinated only to 3020.0 BAU/mL in hybrid immunized subjects (*p*-value < 0.0001). Over time, a significant decline in the levels of neutralizing antibodies was found. Depending on the subpopulation analyzed, further significant influencing factors included sex assigned at birth, disease severity, chronic diseases, and medication. A hybrid immunization resulted in more robust immune responses. Nevertheless, there were multiple other factors impacting these responses. This knowledge should be included in future vaccination strategies and serve as a guide in the development of personalized medicine.

## 1. Introduction

To combat the coronavirus disease 2019 (COVID-19) pandemic and thereby avoid or reduce long-term and strong immediate effects of COVID-19, the development of severe acute respiratory syndrome coronavirus type 2 (SARS-CoV-2) vaccines was initiated almost immediately after the start of the outbreak in 2019 [1]. In Austria, the most commonly used vaccines were the mRNA vaccine types Comirnaty—BNT162b2 (BioNTech Manufacturing GmbH, Mainz, Germany) and Spikevax—mRNA-1273 (Moderna Biotech Spain, S.L., Madrid, Spain), as well as the adenoviral vector-based vaccines Vaxzevria—ChAdOx1 (Astra Zeneca AB, Sodertalje, Sweden) and Jcovden—Ad26.COV2.S (Janssen-Cilag International NV, Beerse, Belgium). These vaccines were approved using fast-track clinical approval. The efficacy of the above-mentioned SARS-CoV-2 vaccines regarding the ability to induce an adaptive immune response in form of specific immunoglobulin G (IgG) antibodies against the receptor-binding domain of the spike protein of SARS-CoV-2 was shown for all vaccines [2,3,4,5].

Seroprevalence studies are of great importance to understand the true infection rates, vaccination status, and other indicators for the immunity of a population. In December 2021, the UNITY Studies together with the World Health Organization (WHO) published a systematic review and meta-analysis of standardized population-based seroprevalence data on the global epidemiology of the SARS-CoV-2 infection from January 2020–October 2021 [6]. In addition, the WHO UNITY published a standardized protocol, the SEROPREV protocol, as a guideline for further population-based, age-stratified sero-epidemiological investigations. This protocol enables the comparability of various studies to inform about the progress of the pandemic on a global level [7]. Since then, a number of seroprevalence studies have been performed [8,9,10]. However, factors influencing the levels of neutralizing antibodies within a population or within subgroups have not yet been thoroughly defined. For this reason, it is of great importance to dissect the factors affecting varying antibody formation after disease and vaccination in order to allow for adapted and ideally personalized vaccination schemes in the future.

It is also known that the formation of specific antibodies did not protect against breakthrough infections [11,12,13]. Although this is known, the antibody level needed to protect against severe cases of COVID-19 still needs to be determined. Concerning the vaccine-induced antibody response, a decreased immunity over time has been reported, especially for the male population, for people taking immunosuppression, and for persons of older age (65 and older) [14,15,16].

Other virus-related infections or vaccination schemes have shown that the levels of antibodies produced greatly depended on a number of influencing factors [17,18]. Especially during the COVID-19 pandemic, this difference in the protection levels against a virus within a population became apparent. Among such factors, sex assigned at birth, age and smoking habits were shown to correlate significantly with the level of antibodies against the spike protein after vaccination in certain cohorts [19,20,21]. It has been shown that the type of SARS-CoV-2 vaccine affects the level of antibodies [22]. In order to deepen the knowledge on factors influencing SARS-CoV-2-specific antibody formation, we designed a population-based study. In this seroprevalence study, we focused on the immune response following SARS-CoV-2 vaccination and/or COVID-19, by considering aspects of influencing factors. These included the number of vaccinations, the type of vaccine, sex assigned at birth, the body mass index (BMI), age, chronic diseases and permanent medication. For this purpose, we obtained and analyzed data from 2000 subjects (mean age 42.6 years, from Salzburg, Austria), statistically representing the adult Austrian population.

The primary objectives of this study were (1) to assess the effect of a range of influencing factors on the levels of specific IgG antibodies produced as a result of SARS-CoV-2 vaccination alone and in combination with natural infections with SARS-CoV-2 and (2) to assess the kinetics of antibody responses up to 24 weeks in a defined subgroup following the general hypothesis that the antibody level is driven by various individual factors in addition to vaccination. This knowledge is of great importance when aiming at future personalized vaccination schemes.

## 2. Materials and Methods

### 2.1. Study Design and Participants

The study cohort consisted of 2000 participants from the county of Salzburg (Austria) aged 18 years and over. The sample size was calculated using SurveyMonkey to represent the adult Austrian population. Subjects were recruited by sending out invitational emails to various institutes/companies and associations in the county of Salzburg. Designated time slots were available for blood drawing using an online planning tool. These participants either received SARS-CoV-2 vaccination and/or experienced the COVID-19 disease.

All participants signed an informed consent form including a data protection statement. A self-designed questionnaire, including a number of factors potentially influencing the level of antibody production, was filled out by all participants. The questionnaire included anthropometric factors (sex assigned at birth, date of birth, height, weight), information regarding the vaccination (date and type of vaccine as stated in electronic vaccination passes), and medical history (chronic disease, permanent medication, COVID-19 infection including the observed disease severity and symptoms). Chronic diseases included in the questionnaire were Morbus Crohn, colitis ulcerous, high blood pressure, psoriasis arthritis, rheumatoid arthritis, lupus erythematosus, diabetes mellitus 1 and 2, kidney diseases, liver diseases, and thyroid diseases. Permanent medication was recorded by stating the medication that was taken. COVID-19 infection was recorded using the official statement about infection date and quarantine end as well as the subjective observed disease severity and symptoms. Prior infection status of the participants was not confirmed by serology.

In addition, time course measurements of SARS-CoV-2-specific antibodies over a period of 24 weeks at specific timepoints (3, 8, 12, and 24 weeks after their last immunizing event) were performed in a subgroup of subjects (n = 77) either (i) three times vaccinated only (n = 28), (ii) four times vaccinated with previous infection (n = 4), or (iii) convalescent subjects with previous vaccinations (once, twice, or three times vaccinated; n = 45). In the latter group, blood was first drawn 3 weeks after quarantine end, whereas in the other two groups blood was drawn after the third or fourth vaccination, respectively. Quarantine end was defined as 10 days after testing positive for COVID-19 by the polymerase chain reaction (PCR) method. The allocation to the three subgroups resulted from the pandemic infection dynamics. Participants were excluded from analysis if infection occurred during the 24-week monitoring.

### 2.2. Blood Sampling and Antibody Detection

Blood sampling was performed between January 2022 and May 2023 on an individual basis, independent of the date of vaccination or SARS-CoV-2 infection. Blood was obtained in a single 3 mL serum tube (CAT serum clot activator, ref. no. 454095, Greiner Bio-One GmbH, Austria) and was left to stand upright at room temperature for 30 min. The sample was than centrifuged at 1300× *g* for 10 min at 22 °C. The sera were immediately transferred into fresh 2 mL microcentrifuge tubes (SARSTEDT AG & Co. KG, Nümbrecht, Germany) and stored at −20 °C until batch analysis.

Upon valid internal quality control results, the qualitative and quantitative detection of the SARS-CoV-2 spike-protein-specific IgG antibodies was performed in accordance with specifications of “Laboratory diagnostics for Coronavirus SARS-CoV-2” of the Austrian Society for Laboratory Medicine and Clinical Chemistry. External quality controls from the Austrian Association for Quality Assurance and Standardization of Medical and Diagnostic Tests (ÖQUASTA) were included over the duration of this study.

SARS-CoV-2 spike-protein-specific IgG antibodies were analyzed using the VITROS Immunodiagnostic Products “Anti-SARS-CoV-2 IgG Quantitative assay” (including Quantitative Reagent Pack and Quantitative Calibrators) on the VITROS ECiQ Immunodiagnostic analytic instrument (Ortho Clinical Diagnostics GmbH, Germany). The chemiluminescence immunoassays were performed following the manufacturers protocol. The measuring range of SARS-CoV-2-specific IgG antibodies ranged from 0 to 4000 BAU/mL.

### 2.3. Statistical Analysis

The demographic data from the questionnaires and the corresponding analytical results were collected in an EXCEL spreadsheet (version 1808, Microsoft, Redmond, WA, USA). Thereafter, data were transferred and statistically analyzed using the IBM SPSS Statistics (version 27, Armonk, NY, USA) and GraphPad Prism (version 9.2.0, GraphPad Software Boston, MA, USA) software. Normality was evaluated using the Kolmogorov–Smirnov test. To determine the differences between antibody levels, either the Mann–Whitney U-test for two-sample comparison or the Kruskall–Wallis test with subsequent pairwise comparison using Dunn’s test were used. Correlation analysis was performed using the Spearman Rho test. Time-course measurements were analyzed using the Friedman test followed by Dunn’s multiple comparison test. Data are presented as median (interquartile range (IQR)) values due to non-normalized data distribution.

## 3. Results

### 3.1. Subject Demographics

**Figure 1:**
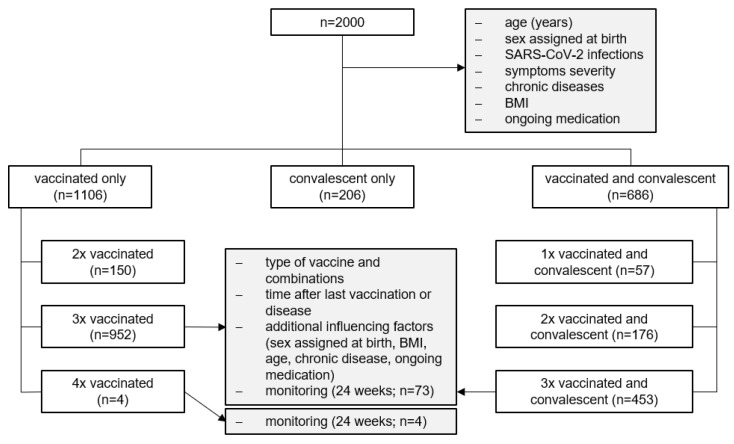
Analysis tree depicting the different study groups.

Between January 2022 and May 2023, 2000 eligible individuals were enrolled in the study and their data were included in the analysis (Figure 1). The population characteristics as well as statistical analysis of the study population are shown in Table 1.

Out of the 2000 subjects, 66.0% were female and 34.0% male. The average age was 42.6 years and the average BMI was 24.8. A total of 556 (27.8%) subjects reported to suffer from chronic diseases, 31.7% (n = 176) of whom had a thyroid disease and 19.4% (n = 108) reported to have a high blood pressure. Other chronic diseases included rheumatoid arthritis, diabetes mellitus type 1 and 2, kidney disease, liver disease, Morbus Crohn, colitis ulcerous, psoriasis arthritis, and lupus erythematosus. These chronic diseases were present within the study population with a rate between 0.2 and 1.3%. A total of 42.1% (n = 234) reported other diseases such as depressions and allergies or multiple chronic diseases. Out of the study population, 599 (30.0%) subjects were prescribed ongoing medication.

No statistical difference of the antibody height was observed between the age categories of 20–40, 41–60, and over 60 years. A significantly higher antibody level was seen in the female compared to the male population and in subjects having had one SARS-CoV-2 infection versus two infections. Subjects experiencing moderate COVID-19 symptoms had a significantly higher antibody titer compared to subjects with no symptoms (Figure S1). No correlation could be found in relation to chronic diseases, BMI, or ongoing medication and the height of the antibody level within the whole population (Table 1).

### 3.2. Effects of the Number of Vaccinations on the Level of IgG

**Figure 2:**
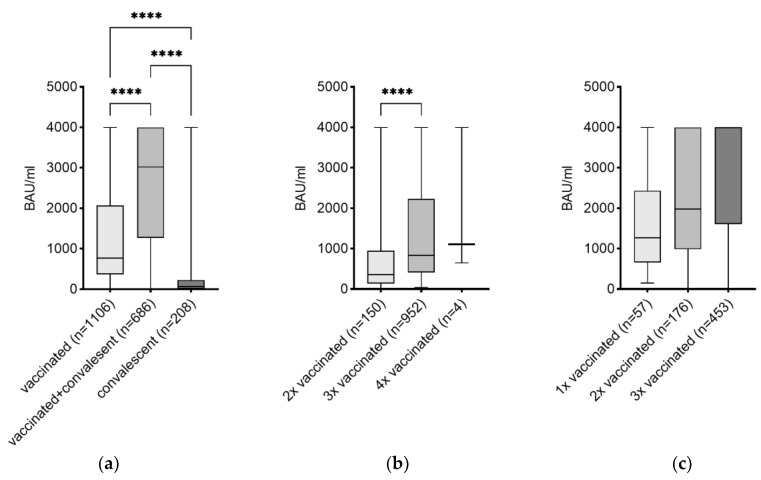
Comparison of the antibody titer. Differences in the antibody titer can be seen within (**a**) vaccinated, vaccinated and convalescent as well as convalescent, (**b**) vaccinated-only subjects, (**c**) vaccinated and convalescent subjects. Antibody titers are depicted as median (IQR). **** *p*-value < 0.0001.

To investigate the influence of vaccination on the level of SARS-CoV-2-specific IgG, the study population was first split into SARS-CoV-2 vaccinated (n = 1106 or 55.3%), vaccinated and convalescent (n = 686 or 34.3%), and SARS-CoV-2 infection convalescent-only (n = 208 or 10.4%) subjects. The highest median IgG antibody level (3020.0 BAU/mL) was detected in subjects with a hybrid SARS-CoV-2 immunity compared to subjects with SARS-CoV-2 vaccination only (770.5 BAU/mL) and convalescence only (65.6 BAU/mL) (Figure 2a). To further determine the influence of the number of vaccinations, the vaccinated study population was split into twice vaccinated (n = 150 or 13.6%), three times vaccinated (n = 952 or 86.1%), and four times vaccinated (n = 4 or 0.4%). Subjects who received three doses of SARS-CoV-2 vaccines had a significantly higher median titer (835.5 BAU/mL) compared to those with two doses of vaccines (361.5 BAU/mL) (Figure 2b). The same was performed in the group of vaccinated and convalescent subjects, whereby 57 subjects (8.3%) were one-time vaccinated and convalescent, 176 (25.7%) were two-times vaccinated and convalescent, and 453 (66.0%) were three-times vaccinated and convalescent. Subjects with infection-inquired immunity and a three-time vaccination had a significantly higher median titer (4000.0 BAU/mL) compared to those with two doses of vaccine (1980.0 BAU/mL) and with one dose of vaccine (1270.0 BAU/mL) (Figure 2c). Descriptive statistics including confidence intervals for data shown in Figure 2 can be found in Supplementary Materials, Table S1.

Further analysis of influencing factors was performed on study populations of three-times vaccinated as well as three-times vaccinated and convalescent due to the highest sample size within these subgroups.

### 3.3. Vaccination Type and Its Combination as Influencing Factor

**Figure 3:**
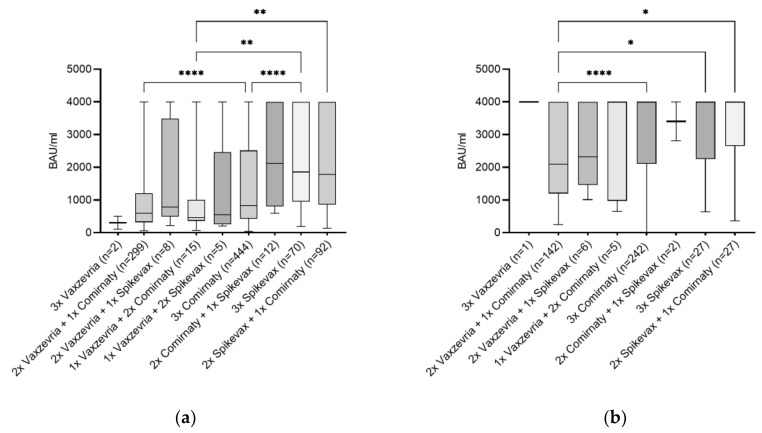
Effect of vaccine types and combinations on the level of SARS-CoV-2 IgG. Within (**a**) three-times vaccinated only and (**b**) three-times vaccinated and convalescent group differences in the IgG titers with respect to different vaccination concepts were observed. Antibody titers are depicted as median (IQR). * *p*-value < 0.05; ** *p*-value < 0.01; **** *p*-value < 0.0001.

To determine the influence of the type of vaccination and vaccination combinations on the level of SARS-CoV-2-specific IgG, the three-times vaccinated group as well as the three-times vaccinated and convalescent group were further divided into groups representing the various different vaccine types and combinations present in the cohort (Figure 3). In both groups, subjects vaccinated with three doses Vaxzevria as well as those immunized with Jcovden, BBIBP-CorV (Sinopharm Chemical Reagent Co, Ltd., Shanghai, China), NVX-CoV2373 (Novavax Inc., Gaithersburg, MD, USA), and Sputnik V (Biocad, Moscow, Russia) were not included in the analysis due to the low number of participants.

Within the three-times vaccinated-only group, the highest antibody median value was reached with the combination of two doses of Comirnaty with one dose of Spikevax (2115.0 BAU/mL) followed by three doses of Spikevax (1855.0 BAU/mL) and the combination of one dose of Comirnaty with two doses of Spikevax (1780.0 BAU/mL) (Figure 3a). For the three-times vaccinated and convalescent group, higher median levels compared to the three-times vaccinated-only group were observed. The highest IgG median (IQR) value being 4000.0 (2650.0–4000.0) BAU/mL was reached with two doses of Spikevax and one dose of Comirnaty, followed by three doses of Spikevax with 4000.0 (2250.0–4000.0) BAU/mL and three doses of Comirnaty with 4000.0 (2105.0–4000.0) BAU/mL (Figure 3b). Descriptive statistics including confidence intervals for data shown in Figure 3 can be found in Supplementary Material, Table S1.

### 3.4. Time as Influencing Factor on IgG Level

To determine the effect of time elapsed from the day of vaccination or last infection to the day of blood drawing, a correlation analysis was performed. First, a time-dependent difference between the height of antibody level in one-, two-, three-, and four-times vaccinated subjects within the vaccinated-only and the hybrid immunized groups (see Figure 2a,b) was analyzed. The increased antibody level in three-times vaccinated subjects could not be explained by factor time since the median timespan between vaccination and blood drawing was significantly higher in three-times vaccinated than in two-times vaccinated subjects in both groups (Figure S2 and Table S2). In addition, time as an influencing factor accounting for the height of the antibody levels seen in Figure 3a,b could be ruled out, since analysis of the time span passed between vaccination and blood drawing revealed no significant difference (except for vaccinations with two doses of Spikevax and one dose of Comirnaty versus three doses of Comirnaty) (Figure S3).

Nevertheless, when analyzing the three-times vaccinated-only group specifically, a significant negative correlation was observed between the level of IgG antibodies and the weeks since last vaccination (r = −0.465; *p*-value < 0.0001). For the group of three-times vaccinated and convalescent individuals a lower, but still significant negative correlation (r = −0.275; *p*-value < 0.0001) was observed between time elapsed since recovery (quarantine end date) and its corresponding IgG level. However, no correlation was seen between the titer and weeks after the third vaccination (r = −0.030; *p*-value 0.529).

**Figure 4:**
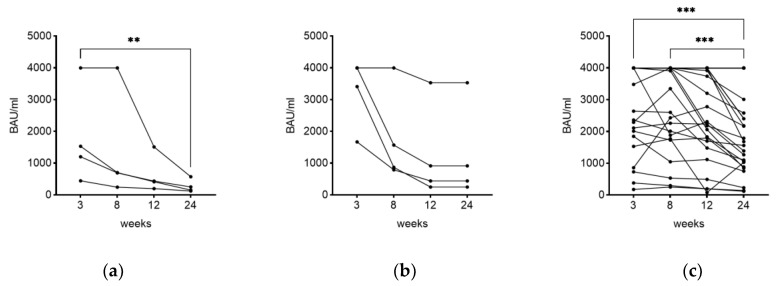
Monitoring the antibody titer over time. Antibody level was determined over a period of 24 weeks at four different timepoints (3, 8, 12, and 24 weeks) in subjects after (**a**) third vaccination only, (**b**) fourth vaccination with previous infection, and (**c**) recovery with previous vaccination. ** *p*-value < 0.01, *** *p*-value < 0.001.

In addition to the decline of the antibody titer as stated above, individual subjects were monitored at four different timepoints (3, 8, 12, and 24 weeks) over a period of 24 weeks. Due to various reasons, a full monitoring could only be achieved for 38 out of 77 participants. Monitoring the level of antibodies over a period of 24 weeks showed a significant decline in measured IgG between the first and last timepoint in subjects being three-times vaccinated without previous infection (*p*-value < 0.01) (Figure 4a). In the group of four-times vaccinated subjects with previous infection, no significant effects could be shown (Figure 4b). Within the group of convalescent subjects with previous vaccinations (once, twice, or three times), a significant correlation was observed between the first and last (*p*-value < 0.001) as well as the second and last measuring timepoints (*p*-value < 0.001) (Figure 4c). Information including all 77 participants can be found in Supplementary Materials, Figure S4. In general, a decrease in antibody levels was observed in all three groups within the 24 weeks.

### 3.5. Additional Influencing Factors

Further analysis was performed to identify additional influencing factors on the antibody titer. Within the group of three-times vaccinated-only individuals, no significant influence could be found of factors sex assigned at birth (*p*-value 0.058), BMI (r = −0.008; *p*-value 0.815), age (r = −0.052; *p*-value 0.110), and chronic diseases (*p*-value 0.106) on the antibody titer. A significantly lower median antibody titer (*p*-value < 0.02) was observed in participants taking medication (716.0 BAU/mL) compared to those with no medication (921.0 BAU/mL). Analysis of the three-times vaccinated group and the convalescent group showed no significant influence of sex assigned at birth (*p*-value 0.201), BMI (r = 0.050; *p*-value 0.287), age (r = 0.085; *p*-value 0.070), and taking medication (*p*-value 0.391) on the antibody titer. However, a significant influence (*p*-value < 0.031) of the factor of chronic diseases could be observed. Post hoc analysis between various types of chronic diseases showed that the antibody titer in rheumatoid arthritis patients was significantly lower compared to that of those with thyroid disease (*p*-value < 0.034) (Figure S5 and Table S3).

## 4. Discussion

In the present study involving 2000 participants aged 18 years and above, various factors, including anthropometric factors, medical conditions, time, and vaccination type, were evaluated in relation to the level of SARS-CoV-2 spike proteinspecific IgG antibodies after SARS-CoV-2 vaccination and/or infection. The study was conducted according to the protocol published by the WHO UNITY [23] to enable high-quality and comparability to other seroprevalence studies. The data presented need to be interpreted considering the possible influence of the following limitations: the measuring range was limited to 4000.0 BAU/mL and the number of subjects within various subgroups differed, which sets a limitation regarding the statistical evaluation.

Within the whole population, a positive correlation between the height of spike protein-specific antibody titers after COVID-19 and the level of disease severity (moderate symptoms compared to none) was shown. This correlation has been previously observed related to severe cases of COVID-19 [24].

A strong influence of the type of vaccine and vaccine combinations on the levels of SARS-CoV-2 spike-protein-specific IgG was observed in COVID-19-convalescent as well as uninfected subjects. The lowest median antibody titer (65.6 BAU/mL) was detected in subjects who were convalescent only. This can be explained by the findings that the SARS-CoV-2 adaptive immunity differs between recovered COVID-19 individuals and vaccinated people [25]. Convalescent individuals develop a broader immune response towards multiple epitopes, whereas vaccinated individuals predominantly develop a strong immune response against spike epitopes [26]. Study participants with SARS-CoV-2 vaccination without COVID-19 had a median antibody level of 770.5 BAU/mL. The highest median antibody level was detected in subjects with hybrid immunization (3020.0 BAU/mL). An infection-acquired immunity compared to the immune response upon vaccination appeared to boost immunity, which is consistent with previous studies [27,28]. This suggests that the convalescent-only group would benefit from COVID-19 vaccinations to increase their antibody titer and thus enhance their protection against SARS-CoV-2.

Our data support the Center for Disease Control and Prevention (CDC) recommendation of a third dose (booster) to retain protection due to the significantly higher antibody levels after three vaccinations. The median SARS-CoV-2 spike-protein-specific antibody level increased with the number of vaccination doses within the vaccinated-only and hybrid immunized subjects. Other studies reported similar observations in their experiments—increased titers with an increase in the number of administered doses of SARS-CoV-2 vaccines [20,29].

Furthermore, vaccination type as well as the combination of vaccines have a significant influence on the level of the antibody titer. Within the three-times vaccinated-only group, subjects with a vaccination combination of two doses of Comirnaty and one dose of Spikevax had the highest median antibody titer followed by those with three doses of Spikevax. Furthermore, within the hybrid immunized group, participants with the vaccine combination of two doses of Spikevax and one dose of Comirnaty had the highest median titer, followed by those with three doses of Spikevax. These results are in line with an existing study on heterologous booster vaccinations, which resulted in higher antibody titers compared to homologous vaccinations [30]. Our results also indicate that vaccinations with mRNA-based vaccines led to a higher antibody response than with the adenoviral vector-based vaccine Vaxzevria, although these numbers were low. This observation confirms the results of Doke et al. and Foddis et al., who also could observe a decline over time; however, in this case, the underlying mechanisms still remain unclear [22,31].

Monitoring the dynamics of the antibody responses during a period of 24 weeks showed that the antibody levels declined over time, which was consistent with previous studies [15,16,29,32], indicating that time is a very strong influencing factor. In addition, a significant negative correlation existed between the number of days since the last immunizing event with the detected antibody levels. In hybrid immunized subjects, this negative correlation was observed for the timespan since the last infection, because the infections occurred mainly after the vaccination event. This reduction in IgG naturally occurs in most cases. The dynamics of infection during the course of the 24-weeks period resulted in a number of drop-outs mainly due to infections which occurred during the time-course measurement. These participants could thus not be included when analyzing the dynamics in antibody response over time. A further limiting factor relates to the number of subjects within the subgroups analyzed. Analysis of additional subgroups should be considered in further studies. In order to monitor the immunological memory against SARS-CoV-2, there is a need to develop novel assays that can be performed in a high-throughput manner to monitor cellular memory.

In the presented study, no correlation was found between the antibody titer and influencing factors like sex assigned at birth, BMI and age within the investigated subgroups (vaccination only and vaccination plus convalescent), confirming the findings of Foddis R. et al. [22]. In contrast, other groups reported a lower SARS-CoV-2 IgG level in older participants as well as in the male group or in subjects with an increased BMI [20,21], which corresponds to the significantly lower level of the antibody titer found in male subjects when taking the whole population (n = 2000) into consideration. Sex is known as a biological variable affecting the functions of the immune system. A number of factors account for these differences including sex hormones, reproductive status, age, and sex chromosome genes, all resulting in different susceptibilities to various diseases by influencing both the adaptive and innate immune responses [33]. In our study, more female (66.0%) than male (34.0%) persons participated, indicating a higher willingness to participate in such studies among women. However, this imbalance sets limitations to the generalizability of the results.

Various common chronic diseases were investigated as influencing factors. Subjects with rheumatoid arthritis showed an extenuated antibody titer. This result is consistent with the fact that individuals with rheumatoid arthritis often receive immunosuppressive therapy. However, an in-depth analysis could not be performed due to low number of subjects within certain groups of chronic diseases. A deeper insight into these specific groups with more participants would be of great interest. Studies have shown that older subjects with diabetes tend to have a weaker antibody response to SARS-CoV-2 vaccination and a lower SARS-CoV-2 IgG antibody titer was observed in people with diabetes mellitus type 2 following Comirnaty vaccination [34,35]. Since correlations and trends towards correlation were found for diseases that affect the hormonal system or hormones as such (male versus female), the analysis of the hormone status could be of interest for future studies.

Within the vaccinated only group, participants who took regular medication had a significantly lower antibody titer. This could be associated with the fact that drugs influence the immune system. For example, anti-depressants can lead to a modification of the immune response [36,37]. Surprisingly, this observation could not be confirmed in the other groups investigated. In addition, due to the various medications taken, an arrangement into medication groups resulted in very small numbers of subjects per group, which could not be statistically analyzed. However, such findings are important with regard to personalized medicine, since defined subgroups may respond divergently due to various different influencing factors. Therefore, further studies should investigate the influence of various medication categories on the immune response towards vaccination.

## 5. Conclusions

This study highlights the superiority of hybrid immunization in promoting SARS-CoV-2 antibody responses. The differences in SARS-CoV-2-specific IgG antibody levels in defined populations, depending on the type of vaccine as well as the combination of vaccines, are emphasized. The factor “time since last vaccination or disease” is a key influencing element. We highlight the need to specifically define the population subgroup being analyzed and call attention to the factors “chronic disease” and “medication” as potential influencing factors in the development of neutralizing antibodies. In addition, we have to point out our study limitations in terms of gender imbalance and a low number of subjects within certain subgroups. Future vaccination development schemes should consider the current literature indicating varying antibody protection levels based on the vaccine types and combinations used. In addition, personalized vaccination schemes should be considered based on medical conditions and factors influencing response to a vaccine. Scientific proof showing the effectiveness of multiple vaccinations can be used by policy makers for education campaigns to increase the acceptance of vaccinations by the public.

## Figures and Tables

**Table 1:** Demographics and overview of the study population.

n = 2000	Mean/SD or n-Number	IgG BAU/mL Median (IQR)	Correlation Coefficient (r/*p*-Value)	*p*-Value (IgG Titer) ^1^
**Age in years**	42.6 (12.6)	1070 (382–3573)	−0.033 (0.141)	0.156
20–40	885	1190 (430–3530)	NA ^2^	NA
41–60	986	962 (345·3–3415)	NA	NA
>60	129	1050 (358.5–4000)	NA	NA
**Sex assigned at birth**	NA	NA	NA	0.018 (female vs. male)
Female	1321	1000 (355–3210)	NA	NA
Male	679	1170 (446–4000)	NA	NA
**SARS-CoV-2 infections**	NA	NA	NA	0.002 (1 inf. ^3^ vs. 2 inf.)
1 infection	849	1900 (484–4000)	NA	NA
2 infections	45	843 (276–1590)	NA	NA
3 infections	1	446	NA	NA
**Symptoms severity**	NA	NA	NA	0.020
None	64	965 (250–3115)	NA	NA
Mild	451	1820 (383–4000)	NA	0.083 (compared to none)
Moderate	349	1910 (629–4000)	NA	0.013 (compared to none)
Severe	32	1790 (487–3823)	NA	NA
Hospitalization	0	NA	NA	NA
Breathing difficulties	47	1910 (1160–4000)	NA	NA
**Chronic diseases**	NA	NA	NA	0.134
Morbus Crohn	3	4000 (769–4000)	NA	NA
Colitis ulcerosa	4	1157 (249–2143)	NA	NA
High blood pressure	108	924 (348–4000)	NA	NA
Psoriasis arthritis	4	4000 (1228–4000)	NA	NA
Rheumatoide arthritis	11	581 (130–986)	NA	NA
Lupus erythematodes	1	4000	NA	NA
Diabetes mellitus type 1	4	1090 (426–3280)	NA	NA
Diabetes mellitus type 2	7	526 (419–1700)	NA	NA
Kidney disease	1	1390	NA	NA
Liver disease	3	176 (2.59–363)	NA	NA
Thyroid disease	176	1065 (338–4000)	NA	NA
Other/multiple	234	1115 (507–4000)	NA	NA
**BMI**	NA	NA	0.039 (0.084)	0.614
Underweight (<18.5)	52	860 (335–2580)	NA	NA
Normal (18.5 <=> 24.9)	1151	1050 (375–3210)	NA	NA
Overweight (25.0 <=> 29.9)	559	1080 (383–4000)	NA	NA
Obese (>30.0)	238	1155 (418–4000)	NA	NA
**Ongoing medication**	599	1080 (381–3920)	NA	0.405

^1^ *p*-value from Mann–Whitney U-test or Kruskal–Wallis test with Dunn’s multiple comparison between groups related to IgG BAU/mL. ^2^ NA = not applicable. ^3^ inf. = infection.

## Data Availability

Data available upon request.

## Supplementary Materials

The following supporting information can be downloaded at: https://www.mdpi.com/article/10.3390/vaccines11101615/s1, Figure S1: Comparison of the antibody titer based on categorization of COVID-19 severity; Table S1: Descriptive statistics of Figure 2a–c and Figure 3a,b; Figure S2: Analysis of the time span in weeks passed between vaccination and blood draw comparing 1×, 2×, 3×, and 4× vaccinated subgroups; Table S2: Analysis of the time span in weeks passed between the date of vaccination and blood draw; Figure S3: Analysis of the time span in weeks passed between vaccination and blood draw comparing vaccination combinations; Figure S4: Monitoring the antibody titer over time; Figure S5: Antibody titer (BAU/mL) of the various disease types within the three-times vaccinated and convalescent group; Table S3: Pairwise comparison of chronic diseases in the three-times vaccinated and convalescent group.
