# Supplementary material for: Factors Affecting SARS-CoV-2 IgG Production after Vaccination and/or Disease: A Large-Scale Seroprevalence Study
Source: Vaccines (Basel). 2023 Oct 19;11(10):1615. doi: 10.3390/vaccines11101615 (PMC10611123; doi:10.3390/vaccines11101615)
Supplement: Supplementary file 1 [file vaccines-11-01615-s001.zip › vaccines-2646936-supplementary.pdf]

Table of contents

Figure S1 Comparison of the antibody titer based on categorization of COVID-19 severity 2

Table S1 Descriptive statistics of figures 2a, b, c, and 3a, b 2

Figure S2 Analysis of the time span in weeks passed between vaccination and blood drawing comparing 1x, 2x, 3x, and 4x vaccinated subgroups 3

Table S2 Analysis of the time span in weeks passed between the date of vaccination and blood drawing 4

Figure S3 Analysis of the time span in weeks passed between vaccination and blood drawing comparing vaccination combinations 5

Figure S4 Monitoring the antibody titer over time 5

Figure S5 Antibody titer (BAU/ml) of the various disease types within the 3x vaccinated and convalescent group 6

Table S3 Pairwise comparison of chronic diseases in the 3x vaccinated and convalescent group 6

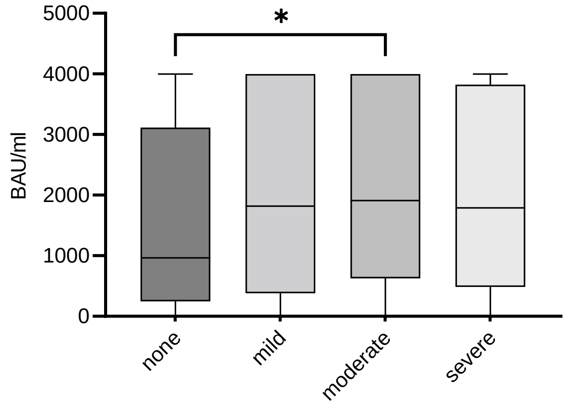

Figure S1. Comparison of the antibody titer based on categorization of COVID-19 severity. Antibody titer (BAU/ml) is compared between subjects with no symptoms, mild-, moderate-, and severe- symptoms. * p-value < 0.05

Table S1 Descriptive statistics of figures 2a, b, c, and 3a, b.

								95% CI of median		
	Number of values	Minimum (BAU/ml)	25% Percentile (BAU/ml)	Median (BAU/ml)	75% Percentile (BAU/ml)	Maximum (BAU/ml)	Range (BAU/ml)	Actual confidence level (%)	Lower confidence limit (BAU/ml)	Upper confidence limit (BAU/ml)
**Figure 2a**										
vacc.[1]	1106	0.00	367.80	770.50	2070.00	4000.00	4000.00	95.00	711.00	835.00
vacc.+conv.[2]	686	0.00	1268.00	3020.00	4000.00	4000.00	4000.00	95.71	2610.00	3730.00
conv.	208	0.00	12.38	65.60	228.80	4000.00	4000.00	95.59	39.10	100.00
**Figure 2b**										
2x vacc.	150	0.00	138.30	361.50	953.30	4000.00	4000.00	95.91	261.00	524.00
3x vacc.	952	40.50	416.00	835.50	2230.00	4000.00	3960.00	95.20	772.00	914.00
4x vacc.	3	648.00	648.00	1110.00	4000.00	4000.00	3352.00	75.00	648.00	4000.00
**Figure 2c**										
1x vacc.	57	154.00	661.50	1270.00	2435.00	4000.00	3846.00	96.69	856.00	1780.00
2x vacc.	177	0.00	986.00	1980.00	4000.00	4000.00	4000.00	96.50	1600.00	2730.00
3x vacc.	453	15.00	1610.00	4000.00	4000.00	4000.00	3985.00	95.17	3610.00	4000.00
**Figure 3a**										
3x Vax. [3]	2	104.00	104.00	304.50	505.00	505.00	401.00	50.00	104.00	505.00
2x Vax. + 1x Com.[4]	299	55.90	318.00	595.00	1210.00	4000.00	3944.00	95.09	530.00	672.00
2x Vax. + 1x Spik.[5]	8	218.00	492.50	786.00	3490.00	4000.00	3782.00	99.22	218.00	4000.00

1x Vax. + 2x Com.	15	70.90	366.00	459.00	1010.00	4000.00	3929.00	96.48	366.00	1010.00
1x Vax. + 2x Spik.	5	208.00	259.50	546.00	2460.00	4000.00	3792.00	93.75	208.00	4000.00
3x Com.	444	40.50	423.00	831.50	2515.00	4000.00	3960.00	95.88	747.00	956.00
2x Com. + 1x Spik.	12	594.00	807.00	2115.00	4000.00	4000.00	3406.00	96.14	762.00	4000.00
3x Spik.	70	192.00	951.50	1855.00	4000.00	4000.00	3808.00	95.86	1220.00	3630.00
2x Spik. + 1x Com.	92	140.00	862.50	1780.00	4000.00	4000.00	3860.00	95.30	1410.00	2790.00
**Figure 3b**										
3x Vax.	1	4000.00	4000.00	4000.00	4000.00	4000.00	0.00	5.00		
2x Vax. + 1x Com.	142	248.00	1198.00	2100.00	4000.00	4000.00	3752.00	96.45	1740.00	2720.00
2x Vax. + 1x Spik.	6	1010.00	1460.00	2315.00	4000.00	4000.00	2990.00	96.88	1010.00	4000.00
1x Vax. + 2x Com.	5	646.00	978.00	4000.00	4000.00	4000.00	3354.00	93.75	646.00	4000.00
3x Com.	242	15.00	2105.00	4000.00	4000.00	4000.00	3985.00	95.39	4000.00	4000.00
2x Com. + 1x Spik.	2	2810.00	2810.00	3405.00	4000.00	4000.00	1190.00	50.00	2810.00	4000.00
3x Spik.	27	634.00	2250.00	4000.00	4000.00	4000.00	3366.00	98.08	2260.00	4000.00
2x Spik. + 1x Com.	27	356.00	2650.00	4000.00	4000.00	4000.00	3644.00	98.08	2890.00	4000.00

[1] vacc.=vaccinated, [2] conv.=convalescent, [3] Vax.=Vaxzevria, [4] Com.=Comirnaty, [5] Spik.=Spikevax

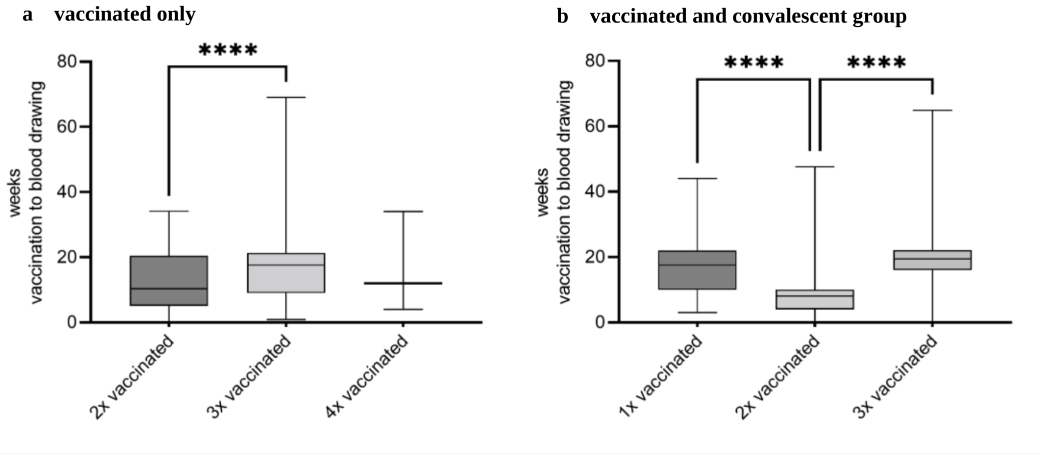

Figure S2. Analysis of the time span in weeks passed between vaccination and blood drawing comparing 1x, 2x, 3x, and 4x vaccinated subgroups. Within the (a) vaccinated only and (b) hybrid immunized group, the weeks passed between vaccination date and date of blood drawing are depicted for the number of vaccinations. **** p-value < 0.0001

Table S2. Analysis of the time span in weeks passed between the date of vaccination and blood drawing. Statistical analyses using descriptive analysis and Kruskal Wallis test with Post-hoc analysis were performed within the (a) vaccinated only and (b) hybrid immunized group comparing 1x, 2x, 3x, and 4x vaccinated subgroups.

a) Vaccinated only subgroup

		Weeks after 2nd vaccination	Weeks after 3rd vaccination	Weeks after 4th vaccination
Median		10.4	17.6	12.0
Minimum		0.1	0.9	4.0
Maximum		34.1	69.0	34.0
Percentile	25	5.1	9.0	4.0
	50	10.4	17.6	12.0
	75	20.5	21.3	34.0

Kruskal Wallis	P-value < 0.000
Pairwise comparison with Bonferroni correction:	
Weeks after 2nd vaccination vs. Weeks after 3rd vaccination	0.000
Weeks after 2nd vaccination Weeks after 4th vaccination	1.000
Weeks after 3rd vaccination vs Weeks after 4th vaccination	1.000

b) Vaccinated and convalescent subgroup

		Weeks after 1st vaccination	Weeks after 2nd vaccination	Weeks after 3rd vaccination
Median		20.7	8.0	19.4
Minimum		4.1	0.0	0.0
Maximum		45.7	47.6	64.9
Percentile	25	13.7	4.0	16.0
	50	20.7	8.0	19.4
	75	24.1	10.0	22.1

Kruskal Wallis	P-value < 0.000
Pairwise comparison with Bonferroni correction:	
Weeks after 1st vaccination vs. Weeks after 2nd vaccination	0.000
Weeks after 2nd vaccination vs. Weeks after 3rd vaccination	0.000
Weeks after 1st vaccination vs Weeks after 3rd vaccination	1.000

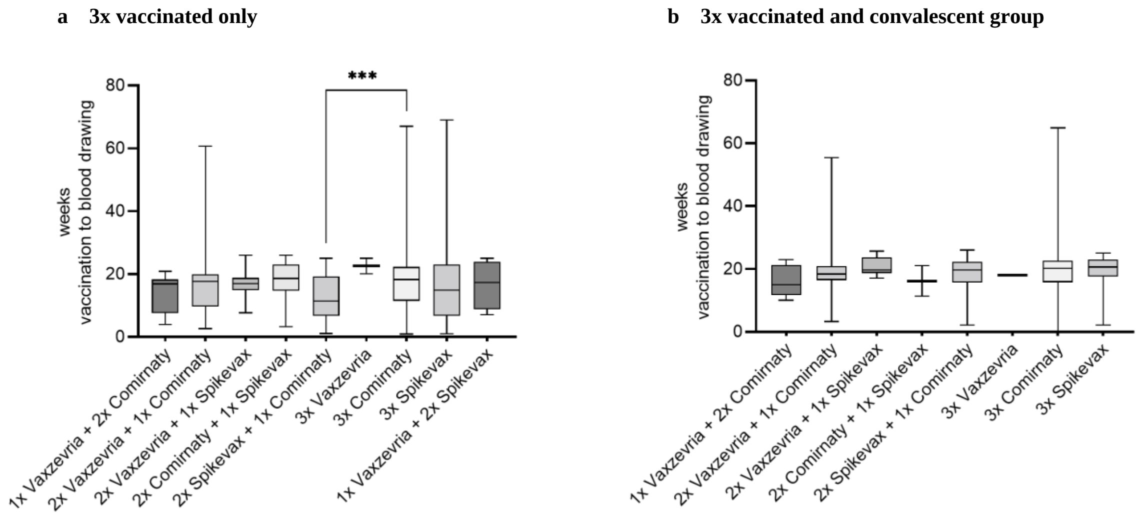

Figure S3. Analysis of the time span in weeks passed between vaccination and blood drawing comparing vaccination combinations. Within the (a) 3x vaccinated only and (b) hybrid immunized group, the weeks passed between vaccination date and date of blood drawing are depicted for the vaccination combinations. *** p-value < 0.001

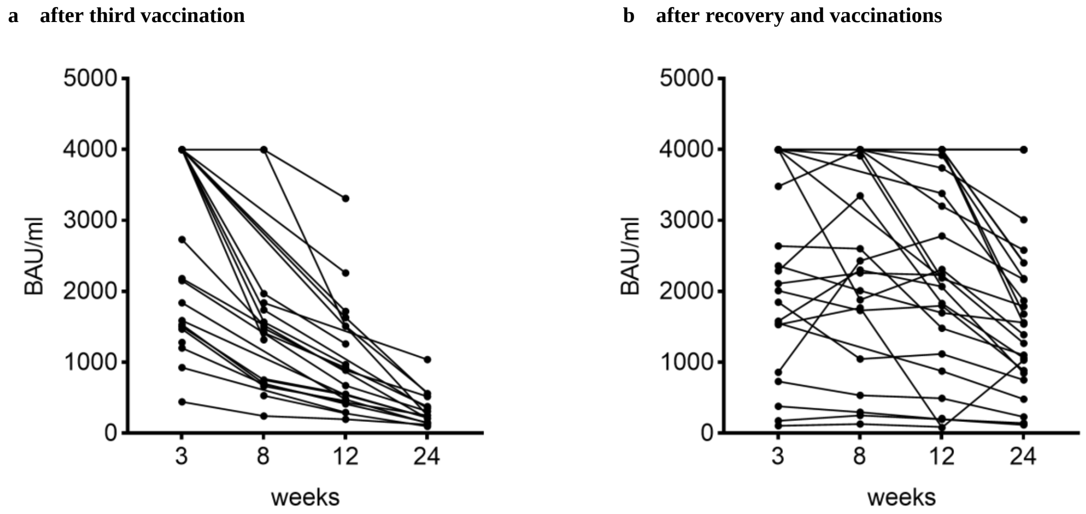

Figure S4. Monitoring the antibody titer over time. Antibody level was determined over a period of 24 weeks at four different timepoints (3, 8, 12, and 24 weeks) in subjects after (a) third vaccination only and (b) recovery and previous vaccinations. Kruskal-Wallis test with Dunn’s post hoc test. *** p-value < 0.001, **** p-value < 0.0001

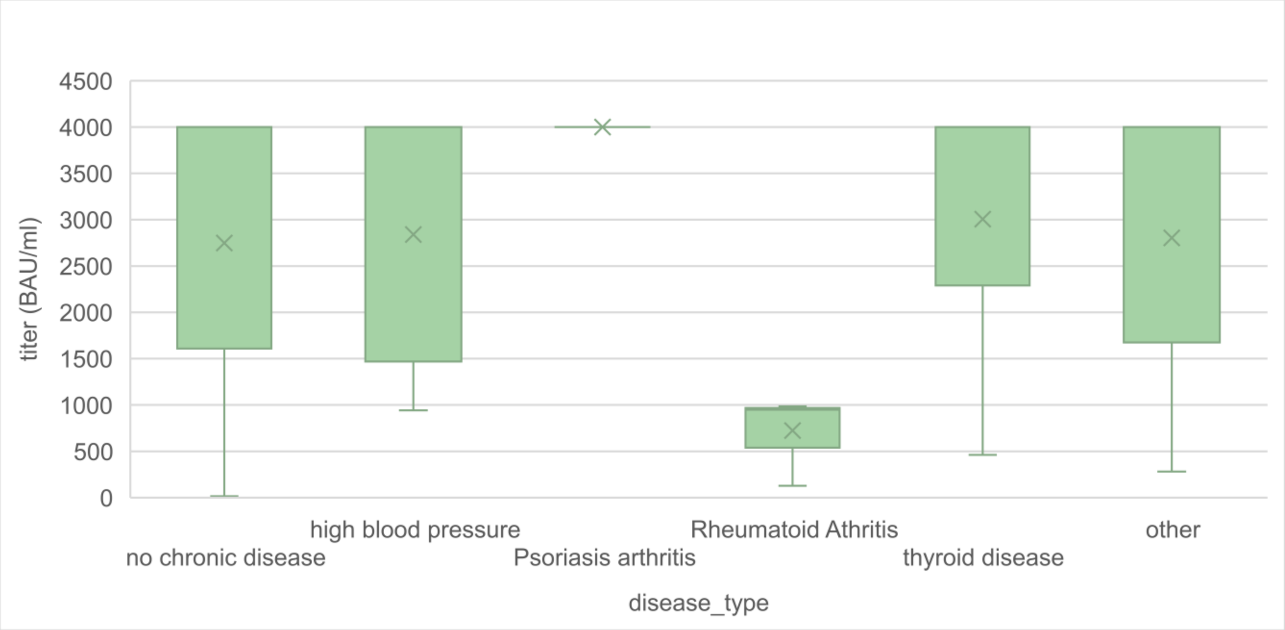

Figure S5. Antibody titer (BAU/ml) of the various disease types within the 3x vaccinated and convalescent group.

Table S3 Pairwise comparison of chronic diseases in the 3x vaccinated and convalescent group.

	Sig.	Corrected. Sig.*
rheumatoid arthritis-no chronic disease	0.010	0.144
rheumatoid arthritis-other	0.008	0.119
rheumatoid arthritis-high blood pressure	0.004	0.055
rheumatoid arthritis-thyroid disease	0.002	0.034
rheumatoid arthritis-psoriasis arthritis	0.034	0.509
no chronic disease-other	0.992	1.000
no chronic disease-high blood pressure	0.327	1.000
no chronic disease-thyroid disease	0.168	1.000
no chronic disease-psoriasis arthritis	0.367	1.000
other-high blood pressure	0.259	1.000
other-thyroid disease	0.081	1.000
other-psoriasis arthritis	0.364	1.000
high blood pressure-thyroid disease	0.888	1.000
high blood pressure-psoriasis arthritis	0.518	1.000
thyroid disease-psoriasis arthritis	0.538	1.000

* after Bonferroni correction
